# Lower Level of* Bacteroides* in the Gut Microbiota Is Associated with Inflammatory Bowel Disease: A Meta-Analysis

**DOI:** 10.1155/2016/5828959

**Published:** 2016-11-24

**Authors:** Yingting Zhou, Fachao Zhi

**Affiliations:** Guangdong Province Key Laboratory of Gastroenterology, Department of Gastroenterology, Nanfang Hospital, Guangzhou, China

## Abstract

*Background and Aims.* Multiple studies have reported associations between inflammatory bowel disease (IBD) and the flora disequilibrium of* Bacteroides*. We performed a meta-analysis of the available data to provide a more precise estimate of the association between* Bacteroides* level in the gut and IBD.* Methods.* We searched PubMed/MEDLINE, EMBASE, Cochrane Library, Wiley Library, BIOSIS previews, Web of Science, CNKI, and ScienceDirect databases for published literature on IBD and gut microbiota from 1990 to 2016. Quality of all eligible studies was assessed using the Newcastle-Ottawa Quality Assessment Scale (NOS). We compared the level of* Bacteroides* in IBD patients with that in a control group without IBD, different types of IBD patients, and IBD patients with active phase and in remission.* Results.* We identified 63 articles, 9 of which contained sufficient data for evaluation. The mean level of* Bacteroides* was significantly lower in Crohn's disease (CD) and ulcerative colitis (UC) patients in active phase than in normal controls. The level of* Bacteroides* in remission CD and UC patients was much lower than patients in the control group.* Bacteroides* level was even lower in patients with CD and UC in active phase than in remission.* Conclusions.* This analysis suggests that lower levels of* Bacteroides* are associated with IBD, especially in active phase.

## 1. Introduction

Inflammatory bowel disease (IBD) which includes Crohn's disease (CD), ulcerative colitis (UC), and indeterminate colitis (IC) [1] is a chronic relapsing inflammatory disorder of the gastrointestinal tract [2] of unclear etiology. One hypothesis is that the inflammation results from altered microbiota in a genetically susceptible host [3].

There are 160 major bacteria among the 1,000 to 1,150 species of bacteria which colonize the human intestinal tract. Two dominant microflora in the human distal gut,* Bacteroides* and Firmicutes phyla, account for 90% of the bacterial flora [4]. Microflora which play an important physiological role in the adult colon are* Bacteroides fragilis*,* Bifidobacterium*,* Bacteroides*, and a variety of anaerobic Gram-positive cocci. Intestinal flora in healthy individuals can demonstrate significant variety. As individuals age, their intestinal microbial flora tend to become more similar [5]. There is a healthy balance of microflora in the gastrointestinal tract in normal individuals. This balance is disrupted in disease.

Many studies have related* Bacteroides* to the development of IBD.* Bacteroides* is a Gram-negative, nonspore forming, obligate anaerobic bacteria normally found in the human intestines, mouth, upper respiratory tract, and genital tract [6].* Bacteroides* expresses polysaccharide A, which can induce regulatory T cell growth and cytokine expression that are protective against colitis [7].

We performed a meta-analysis of case-control studies to assess role of* Bacteroides* in IBD.

## 2. Methods

### 2.1. Data Sources and Searches

A literature search was performed using the Preferred Reporting Items for Systematic Reviews and Meta-Analysis (PRISMA) criteria [8] and Meta-Analysis of Observational Studies in Epidemiology (MOOSE) [9] in the following databases: PubMed/MEDLINE, Cochrane Library, ScienceDirect, EMBASE, BIOSIS previews, Web of Science, CNKI, and Wiley Library. The search period was January 1, 1990, through June 30, 2016. Multiple search strategies were used and keywords were used in preference to MeSH terms to increase the sensitivity of our search. The following key words were used: inflammatory bowel disease, IBD, Crohn's disease, CD, ulcerative colitis, UC, indeterminate colitis, IC, flora disequilibrium, and* Bacteroides*.

An expanded search was done using Google Scholar and by contacting authors of selected articles. Conference abstracts and the bibliography of selected articles were also selected to assure that no relevant studies were missed. If two or more studies shared study populations or more than one article reported the same clinical trial, the publication with more information was selected. Professors Bo Jiang and Yang Bai from the Institute of Digestive Diseases, Nanfang Hospital were contacted as the local experts in the field of gut microbiota.

### 2.2. Study Selection

Studies included for analysis (a) were case-control studies; (b) had the* Bacteroides* level in the intestines determined; (c) were published articles or meeting abstracts; (d) compared the level of* Bacteroides* in patients with IBD and without IBD; (e) were published reports with enough data to analyze differences between the IBD and control groups; (f) were published in English or Chinese.

Two reviewers (YT.Z and FC.Z) independently assessed the quality of all eligible studies using the Newcastle-Ottawa Quality Assessment Scale (NOS) (http://www.ohri.ca/programs/clinical_epidemiology/oxford.asp) for case-control studies. The NOS uses a “stars” rating system to judge quality including selection of the study population, comparability, and exposure assessment. Scores were ranged from 0 (the lowest) to 9 (the highest). Studies with a score ≥ 7 were considered to be of high quality. The quality of each study was awarded stars independently by the same two reviewers (Table 3).

Data was extracted from each qualified study, including the study design, first author's last name, and publication year. Discrepancies between the two authors were dealt with by a consensus meeting with all authors and discussion with our local experts.

### 2.3. Statistic Analysis

Three primary analyses were performed. Different types of IBD patients'* Bacteroides* levels were compared with a control group. The level of* Bacteroides* in patients with CD, UC, and IC were also compared. Patients with active CD or UC and patients with CD or UC in remission were compared. Heterogeneity between studies was assessed using both the *χ* ^2^ test with a *P* value < 0.05 and the inconsistency index (*I* ^2^) with a cut-off of ≥50%.

Statistical analyses were performed using SPSS version 13.0 (SPSS Corporation, Chicago, USA) and Revman version 5.0 (The Cochrane Collaboration, Oxford, UK).

## 3. Results

63 studies were initially identified. Twenty-one studies were excluded with animal experiments, twenty studies were excluded with irrelevant experiments, and eight reviews were excluded. After that, fourteen studies potentially relevant were further screened. One study was excluded because* Bacteroides* was acquired from the gums [10]. Four studies were excluded with data that was inappropriate for analysis [11–14].

**Figure 1:**
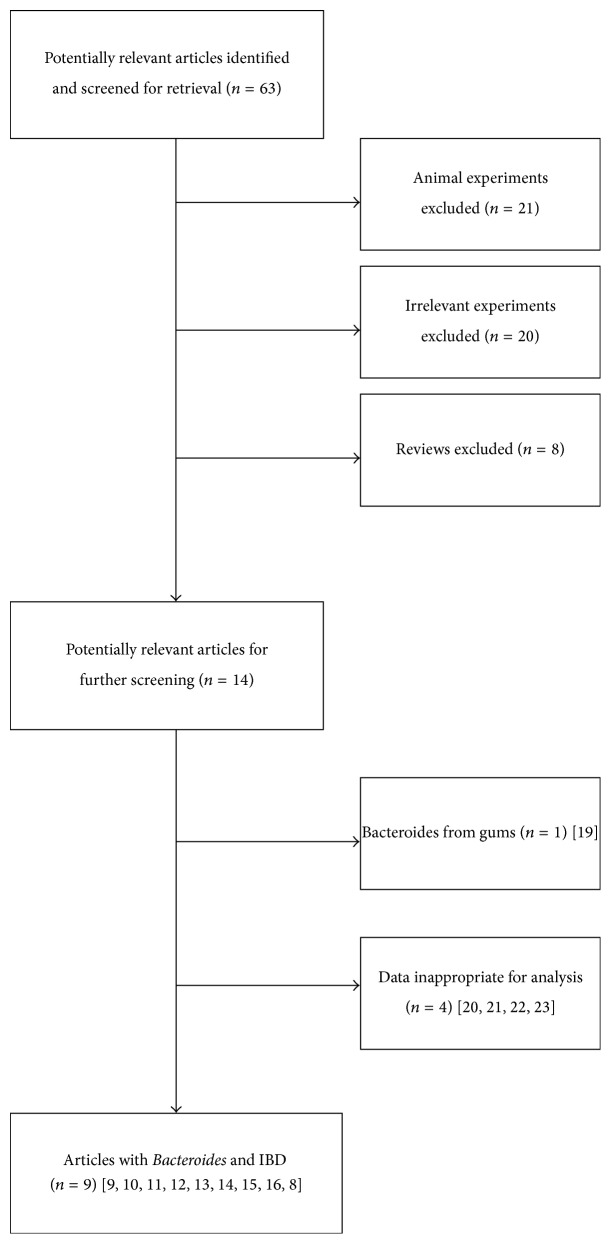
Flow diagram of the study selection process for this meta-analysis.

Finally, 9 studies evaluating 706 patients met the inclusion criteria (Figure 1) [15–23]. Six articles reported continuous data and three reported dichotomous data (Table 1).

The baseline characteristics of all qualified studies are presented in Table 1. Six studies [15, 19–23] included the mean ± standard deviation* Bacteroides* level in different types of IBD patients. This data was used to calculate differences with 95% CIs. Reports with different methods used to measure* Bacteroides* (T-RFLD [15, 23], Real-Time Quantitative PCR [22], conventional culture [20], and FISH [19, 21]) were analyzed separately (Table 2(a)). Three publications [17, 18, 22] with dichotomous data were analyzed using 95% CIs (Table 2(b)). Estimate of the study quality scores from NOS system was showed in Table 3.

The* Bacteroides* level was evaluated for 12 groups, CD versus control group, UC versus control group, IC versus control group, CD versus UC, CD versus IC, UC versus IC, active CD versus remission CD, active UC versus remission UC, active CD versus control group, and active UC versus control group. The active phase of CD was defined using a Crohn's disease activity index (CDAI) >150 and active UC using a Clinical Activity Index (CAI) >5. All patients were diagnosed by endoscopy. Continuous data and dichotomous data were analyzed separately.

### 3.1. Continuous Data

#### 3.1.1. IBD Patients versus Control Group

The amount of* Bacteroides* present in the gut of patients with Crohn's disease was compared to that of a normal control group. Four studies were qualified, with the method of T-RFLP, Real-Time Quantitative PCR, conventional culture, and FISH. Real-Time Quantitative PCR (95% CI: −1.94, −0.19, *P* < 0.01, and Std Mean Difference: −1.42) and conventional culture (95% CI: 0.06, 0.89, *P* = 0.03, and Std Mean Difference: 0.47) both demonstrated statistically significant difference of* Bacteroides* between IBD patients and normal healthy patients. Patients with ulcerative colitis were compared to a normal control group for amount of* Bacteroides* present in the gut. Real-Time Quantitative PCR (95% CI: −1.11, 0.42, *P* < 0.01, and Std Mean Difference: −0.77) and FISH (95% CI: 0.46, 1.57, *P* < 0.01, and Std Mean Difference: 1.01) both demonstrated statistically significant difference of* Bacteroides* between IBD patients and in normal controls. No differences were found in patients with indeterminate colitis.

**Figure 2:**
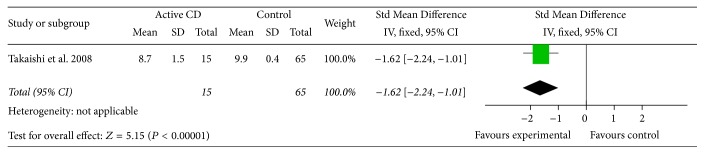
Forest plot of active CD versus control group.

**Figure 3:**
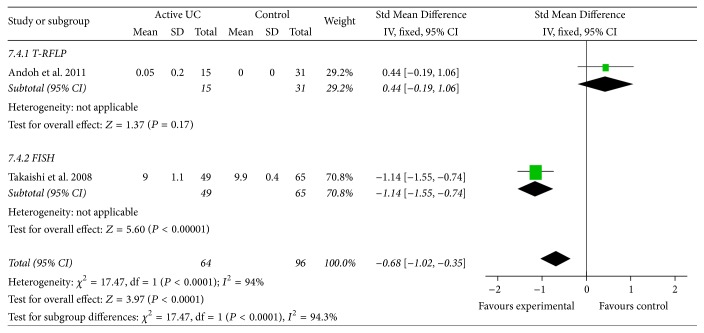
Forest plot of active UC versus control group.

Patients with active Crohn's disease were compared to a normal control group for amount of* Bacteroides*. One study included with the method of Real-Time Quantitative PCR (95% CI: −2.24, −1.01, *P* < 0.01, and Std Mean Difference: −1.62) demonstrated less* Bacteroides* present in the gut of active CD patients than in normal controls (Figure 2). Patients with active ulcerative colitis were compared to a normal control group for amount of* Bacteroides*. Two studies including T-RFLP and FISH test for the overall effect (95% CI: −1.12, −0.35, *P* < 0.01, and Std Mean Difference: −0.68) demonstrated less* Bacteroides* present in their gut than in normal controls (Figure 3).

Patients with Crohn's disease in remission were compared to a normal control group for amount of* Bacteroides* with two studies in Real-Time Quantitative PCR and T-RFLP (Tables 5(a) and 5(b)). Statistically significant difference was found in the amount of* Bacteroides* of CD in remission and normal control patients (95% CI: −1.65, −0.15, *P* = 0.02, and Std Mean Difference: −0.54). Patients with ulcerative colitis in remission were compared to a normal control group with the same two studies of Real-Time Quantitative PCR and T-RFLP. Patients with UC (95% CI: −0.98, −0.09, *P* = 0.02, and Std Mean Difference: −0.90) had less* Bacteroides* present in the gut than normal controls.

#### 3.1.2. Different Types of IBD Patients

Both conventional culture (95% CI: 0.04, 0.70, *P* = 0.03, and Std Mean Difference: 0.37) and FISH (95% CI: 0.1, 1.31, *P* = 0.02, and Std Mean Difference: 0.71) identified significantly lower* Bacteroides* levels of CD patients than in IC patients (Tables 5(c) and 5(d)). No difference was found in the* Bacteroides* level in the gut of CD versus UC and UC versus IC patients.

#### 3.1.3. IBD Patients with Active Phase and in Remission

**Figure 4:**
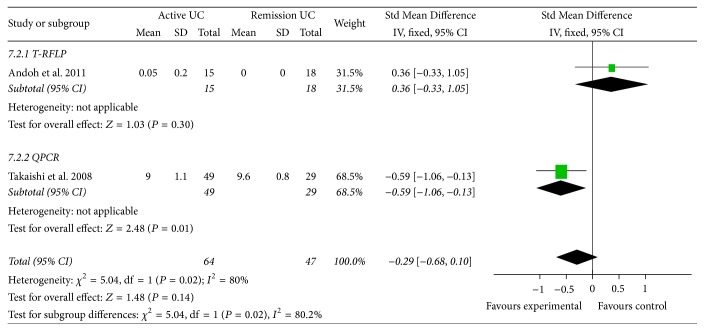
Forest plot of UC in active phase versus in remission phase.

Patients with UC in active phase had less* Bacteroides* in the gut than in remission phase (95% CI: −0.98, 0.09, *P* = 0.02, and Std Mean Difference: −0.29) (Figure 4). Significant difference was also found in the amount of* Bacteroides* of active CD and remission CD patients (95% CI: −1.48, 0.28, *P* < 0.01, and Std Mean Difference: −0.60).

### 3.2. Dichotomous Data

No difference was found in the amount of* Bacteroides* of any groups (CD versus control, UC versus control, CD versus UC, CD versus IC, UC versus IC, and IC versus control). There was no evidence of heterogeneity amongst the groups. Comparisons of IC versus control, active CD versus remission CD, active UC versus remission UC, active CD versus control group, active UC versus control group, remission CD versus control group, and remission UC versus control group were not performed as there were not enough patients to perform this analysis (Table 4).

## 4. Discussion

The relationship between intestinal flora and IBD is well described [24, 25].* E. coli* [13],* Lactobacillus* [26, 27], and* Bacteroides* have been reported to be linked to IBD [28]. Our meta-analysis compared the level of* Bacteroides* in IBD patients with that in normal controls. Patients with active and inactive disease were also evaluated.

A lower level of* Bacteroides* was demonstrated with Real-Time Quantitative PCR in CD and UC patients than in healthy controls, especially CD and UC patients with active disease. UC patients in remission also had a lower level of* Bacteroides* than controls. Patients with active UC had lower* Bacteroides* levels than patients in remission.

CD and UC patients had a higher level of* Bacteroides* in the gut than control patients in FISH and conventional culture studies. These methods also demonstrated a higher level of* Bacteroides* in the gut of CD patients than in IC patients. The level of* Bacteroides* in active IC patients was not reported.

CD and UC patients had lower levels of* Bacteroides* than normal controls in Real-Time Quantitative PCR studies, but higher levels of* Bacteroides* than normal controls in FISH and conventional culture studies. Real-Time Quantitative PCR [22] fecal samples were mostly obtained from Asian patients, while FISH and conventional culture fecal samples were mostly from European patients. This finding suggests* Bacteroides* levels may be different in different ethnic groups.

Heterogeneity was found using a fixed effects model in the pooled UC versus control group, IC versus control group, and CD versus IC group. All these reports used FISH to determine* Bacteroides* levels. Similar findings were observed using a random effects model. Heterogeneity was attributed to two European reports, that of Sokol et al. [19] (using fecal samples) and that of Swidsinski et al. [21] (using tissue samples).

A meta-analysis is inherently limited by the studies included. In this study, many of the reports were descriptive in nature and had small sample sizes. Only two of the 6 studies reported continuous data that could be used to calculate* Bacteroides* levels in patients with active disease and disease in remission. Therefore, significant heterogeneity was found between the studies when data was pooled. We thought the source of the heterogeneity maybe resulted from the difference of methods to determine* Bacteroides* and the age. While the included publications used different methods and had small sample sizes and different patient populations, they all reached the conclusion that* Bacteroides* level was associated with IBD, especially in patients with active disease.

This meta-analysis of observation studies supports the finding of low* Bacteroides* levels in patients with IBD. Despite the differences in methods, our meta-analysis demonstrated that low levels of* Bacteroides* were present in IBD patients with active disease. This finding may be useful in the treatment and the etiology research of IBD patients. Prospective trials are needed to confirm the results of this meta-analysis.

## Figures and Tables

**Table 1:** Baseline characteristics of the included studies.

	First author								
	Andoh [23]	Andoh [15]	Ashorn [16]	Basset [17]	Kleessen [18]	Sokol [19]	Swidsinski [20]	Swidsinski [21]	Takaishi [22]
Year	2011	2012	2009	2004	2002	2006	2002	2005	2008
Publication type	Full text	Full text	Full text	Full text	Full text	Full text	Full text	Full text	Full text
Data type	Continuous	Continuous	Dichotomous	Dichotomous	Dichotomous	Continuous	Continuous	Continuous	Continuous
Method of measurement	T-RFLP	T-RFLP	ELISA	PCR	FISH	FISH	QPCR^Δ^	FISH	QPCR
Sample evaluated	Fecal	Fecal	Fecal	Tissue	Tissue	Fecal	Fecal	Tissue	Fecal
Total # cases									
CD	31	161	18	11	15	13	54	20	23
UC	31	NA	36	20	22	13	119	20	73
IC	NA	NA	4	4	NA	5	104	20	NA
Control	31	121	13	37	15	13	40	20	65
# patients	93	182	71	72	52	44	317	80	161
Age group	Adult	Adult	Adolescent	Adult	Adolescent adult	Adult	Adolescent adult	Adult	Adolescent adult
% weight	9.0	17.5	3.6	6.9	5.0	4.2	30.5	7.7	15.5

CD = Crohn's disease, UC = ulcerative colitis, IC = indeterminate colitis, and NA = no data available; Δ: QPCR: Real-Time Quantitative PCR.

**(a):** 

	First author					
	Andoh [23]	Andoh [15]	Sokol [19]	Swidsinski [20]	Swidsinski [21]	Takaishi [22]
Year	2011	2012	2006	2002	2005	2008
Method	T-RFLP	T-RFLP	FISH	Conventional culture	FISH	QPCR^Δ^
*Bacteroides* levels in CD patients	0.01 ± 0.05*∗*10^3^	21.6 ± 15.4*∗*10^3^	13.8 ± 11.8	2.0 ± 5.5	70 ± 20	8.9 ± 1.2
*Bacteroides* levels in UC patients	0.02 ± 0.12*∗*10^3^	NA	11.7 ± 11.7	1.6 ± 10	62 ± 25	9.3 ± 1.0
*Bacteroides* levels in IC patients	NA	NA	36.4 ± 22.9	0.64 ± 2.1	40 ± 19	NA
*Bacteroides* levels in control group	0	16.6 ± 5.2*∗*10^3^	12.1 ± 7.0	0.02 ± 0.05	20 ± 11	9.9 ± 0.4

CD = Crohn's disease, UC = ulcerative colitis, IC = indeterminate colitis, and NA = no data available; Δ: QPCR: Real-Time Quantitative PCR.

**(b):** 

	First author		
	Ashorn [16]	Basset [17]	Kleessen [18]
Year	2009	2004	2002
Patients with CD (total)	11 (18)	3 (10)	7 (15)
Patients with UC (total)	15 (36)	4 (18)	8 (22)
Patients with IC (total)	4 (4)	1 (4)	(NA)
Patients in control group (total)	(NA)	8 (32)	6 (15)

CD = Crohn's disease, UC = ulcerative colitis, IC = indeterminate colitis, and NA = no data available.

**Table 3:** Scores from NOS system.

Studies	Comparability				Selection		Exposure			Score
	(1)	(2)	(3)	(4)	(1a)	(1b)	(1)	(2)	(3)	
Andoh et al. 2011 [23]	∗	∗	∗	∗	∗	∗	∗	∗	∗	9
Andoh et al. 2012 [15]	∗	∗	∗	∗	∗	∗	∗	∗	—	8
Ashorn et al. 2009 [16]	∗	—	∗	∗	∗	—	∗	∗	∗	7
Basset et al. 2004 [17]	∗	∗	∗	∗	∗	—	∗	∗	∗	8
Kleessen et al. 2002 [18]	∗	∗	∗	∗	∗	∗	∗	∗	∗	9
Sokol et al. 2006 [19]	∗	—	—	∗	∗	∗	—	∗	—	6
Swidsinski et al. 2002 [20]	∗	∗	∗	∗	∗	—	∗	∗	∗	8
Swidsinski et al. 2005 [21]	∗	∗	—	∗	∗	—	∗	∗	∗	8
Takaishi et al. 2008 [22]	∗	—	—	∗	∗	∗	—	∗	∗	7

Selection: (1) the case definition being adequate; (2) representativeness of the cases; (3) selection of controls, and (4) definition of controls. Comparability: (1a) comparability of cases and controls on the basis of CD, UC, and IC diagnosed of endoscope and pathological section; (1b) study controls for active phase with remission phase with IBD patients with CAI and CDAI. Exposure: (1) ascertainment of exposure; (2) same method of ascertainment for cases and controls; and (3) nonresponse rate.

**Table 4:** Summary of outcomes of included studies with dichotomous data.

Groups compared	OR	95% CI	*P* value	*I* ^2^ (*P*)
CD versus control group	1.3	(0.45, 3.77)	0.63	0% (*P* = 0.98)
UC versus control group	0.86	(0.33, 2.24)	0.75	0% (*P* = 1.0)
CD versus UC	1.8	(0.83, 3.93)	0.14	0% (*P* = 0.9)
CD versus IC	0.46	(0.08, 2.69)	0.39	0% (*P* = 0.32)
UC versus IC	0.25	(0.05, 1.28)	0.1	32% (*P* = 0.22)

CD = Crohn's disease, UC = ulcerative colitis, and IC = indeterminate colitis.

**(a):** 

	Standard Mean Difference	95% CI	*P* value	*I* ^2^ (*P*)
CD versus control group	0.23	(−0.42, 0.88)	0.48	NA
UC versus control group	0.23	(−0.27, 0.73)	0.36	NA
CD versus UC	−0.11	(−0.61, 0.39)	0.67	NA
^*∗∗∗*^Active UC versus remission UC	0.36	(−0.33, 1.05)	0.30	NA
Active UC versus control group	0.44	(−0.19, 1.06)	0.17	NA

CD = Crohn's disease, UC = ulcerative colitis, IC = indeterminate colitis, and NA = not available; ^*∗∗∗*^active UC: CAI > 5.

**(b):** 

	Standard Mean Difference	95% CI	*P* value	*I* ^2^ (*P*)
CD versus control group	−1.42	(−1.94, −0.19)	*P* < 0.001	NA
UC versus control group	−0.77	(−1.11, −0.42)	*P* < 0.001	NA
CD versus UC	−0.38	(−0.85, 0.09)	0.12	NA
^*∗∗*^Active CD versus remission CD	−0.60	(−1.48, 0.28)	*P* < 0.01	NA
^*∗∗∗*^Active UC versus remission UC	−0.29	(−0.98, −0.09)	0.02	NA
Active CD versus control group	−1.62	(−2.24, −1.01)	*P* < 0.001	NA
Active UC versus control group	−0.68	(−1.02, −0.35)	*P* < 0.01	NA
Remission CD versus control group	−0.54	(−1.65, 0.15)	0.02	NA
Remission UC versus control group	−0.90	(−0.98, −0.09)	0.02	NA

CD = Crohn's disease, UC = ulcerative colitis, IC = indeterminate colitis, and NA = not available; ^*∗∗*^active CD: CDAI > 150; ^*∗∗∗*^active UC: CAI > 5; Δ: QPCR: Real-Time Quantitative PCR.

**(c):** 

	Standard Mean Difference	95% CI	*P* value	*I* ^2^ (*P*)
CD versus control group	0.47	(0.06, 0.89)	0.03	NA
UC versus control group	0.18	(−0.18, 0.54)	0.32	NA
CD versus UC	0.04	(−0.28, 0.37)	0.78	NA
CD versus IC	0.37	(0.04, 0.70)	0.03	NA
UC versus IC	0.13	(−0.13, 0.39)	0.34	NA
IC versus control group	0.34	(−0.02, 0.71)	0.07	NA

CD = Crohn's disease, UC = ulcerative colitis, IC = indeterminate colitis, and NA = not available.

**(d):** 

	Standard Mean Difference	95% CI	*P* value	*I* ^2^ (*P*)
CD versus control group	0.17	(−0.6, 0.94)	0.67	NA
UC versus control group	1.01	(0.46, 1.57)	*P* < 0.001	93% (*P* < 0.001)
CD versus UC	0.17	(−0.60, 0.94)	0.66	NA
CD versus IC	0.71	(0.1, 1.31)	0.02	94% (*P* < 0.001)
UC versus IC	0.37	(−0.2, 0.95)	0.20	92% (*P* < 0.001)
IC versus control group	−0.54	(−1.13, 0.06)	0.08	94% (*P* < 0.001)

CD = Crohn's disease, UC = ulcerative colitis, IC = indeterminate colitis, and NA = not available.
